# Ohio State Veterinary Medical Association

**Published:** 1898-03

**Authors:** William H. Gribble

**Affiliations:** Secretary


### OHIO STATE VETERINARY MEDICAL ASSOCIATION.

THIS Association convened for its fifteenth annual session in the parlors of the Hotel Goodale, Columbus, Ohio, on January 12, 1898. The meeting was called to order by President Dr. E. H. Shepard at 7.45 P.M. Roll-call showed a good attendance of members as well as visitors. Minutes of last meeting were read and approved.

Election of officers for the ensuing year resulted as follows: President, Walter Shaw, V.S., Dayton; First Vice-President, W. J. Torrance, D.V.S., Cleveland; Second Vice-President, L. W. Carl, V.S., Columbus; Third Vice-President, S. D. Myers, V.S., Wilmington; Secretary, William H. Gribble, D.V.S., Elyria; Treasurer, T. B. Hillock, V.S., Columbus. Three new members were elected: J. E. Foster, V.S, Coshocton (Ont., 1891); B. J. Schmidt, D.V.S., Wapakoneta (New York, 1892); R. J. Mitchener, V.S., Lebanon (Ont., 1885).

The remainder of the evening was used very beneficially to all, in the reporting of interesting cases, and at 10.30 P.M. adjourned.

The session reconvened at 8 A.M., January 13th, when the Committee on Contagious Diseases and Committee on Veterinary Law rendered reports.

Dr. Kent, of Cadiz, Ohio, exhibited some of the finest specimens of tuberculosis it has ever been our pleasure to see. They consisted of the heart, mesentery, and piece of lung of a cow he had destroyed, one he had been called to see on account of an abortion.

The Auditing Committee reported a balance on hand of $283.04.

A motion was made and carried to appoint a committee of two Columbus veterinarians to watch legislation during the present session of the Legislature. The chair appointed Drs. D. S. White and T. B. Hillock.

The members agreed to meet in semi-annual session in Toledo during July, and then adjourned.

WILLIAM H. GRIBBLE, D.V.S.,
Secretary.

